# Unusual case of pancreatic inflammatory myofibroblastic tumor associated with spontaneous splenic rupture

**DOI:** 10.1186/1749-7922-5-28

**Published:** 2010-11-22

**Authors:** Kamal S Hassan, Hector I Cohen, Fadi K Hassan, Shadi K Hassan

**Affiliations:** 1 Clalit Health Services, Ruth and Bruce Rappaport Faculty of Medicine, Technion, Haifa, Israel; 2 Pathology Department, Western Galilee Hospital, Nahariya, Israel; 3 Sackler Faculty of Medicine, Tel Aviv University, Israel; 4 Internal Medicine Department, Carmel Medical Center, Haifa, Israel

## Abstract

**Background:**

Spontaneous splenic rupture considered a relatively rare but life threatening. The three commonest causes of spontaneous splenic rupture are malignant hematological diseases, viral infections and local inflammatory and neoplastic disorders. We describe a unique and unusual case of inflammatory myofibroblastic tumor of the tail of pancreas presented with massively enlarged spleen and spontaneous splenic rupture.

**Case presentation:**

A 19 years old male patient with no significant past medical history presented to emergency room with abdominal pain and fatigue. Massively enlarged spleen was detected. Hypotension and rapid reduction of hemoglobin level necessitated urgent laparatomy. About 1.75 liters of blood were found in abdominal cavity. A large tumor arising from the tail of pancreas and local rupture of an enlarged spleen adjacent to the tumor were detected. Distal pancreatectomy and splenectomy were performed. To our knowledge, we report the first case of massively enlarged spleen that was complicated with spontaneous splenic rupture as a result of splenic congestion due to mechanical obstruction caused by an inflammatory myofibroblastic tumor of the tail of pancreas. A review of the literature is also presented.

**Conclusion:**

Inflammatory myofibroblastic tumor of the tail of pancreas should be included in the differential diagnosis of the etiological causes of massively enlarged spleen and spontaneous splenic rupture.

## Introduction

The most common causes of splenomegaly are liver diseases (33%), hematologic malignancies (27%), infections (23%), congestion or inflammation (8%), primary splenic diseases (4%) and others (5%) [1]. Cirrhosis, lymphoma, AIDS and endocarditis, congestive heart failure and splenic vein thrombosis considered the most common causes in each variety - respectively [1]. There are only a few conditions that cause massively enlarged spleen including chronic myeloid leukemia, hairy cell leukemia, lymphoma, myelofibrosis, thalassemia major, visceral leishmaniasis, malaria, tropical splenomegaly syndrome, AIDS with Mycobacterium avium complex and Gaucher disease [2]. Spontaneous splenic rupture considered a relatively rare but life threatening. Recently, Renzulli et al reported a systematic review of 845 cases with spontaneous splenic rupture that had been published over more than 28 years [3]. In 84.1 percent of cases a single etiological factor was found. Two underlying pathologies were found in 8.2 percent of cases and three or more etiological factors were found in 0.7 percent of cases. The three commonest causes of spontaneous splenic rupture were malignant hematological diseases, viral infections and local inflammatory and neoplastic disorders. Massively enlarged spleen and spontaneous splenic rupture caused by inflammatory myofibroblastic tumor (IMT) of the tail of pancreas is unusual and had been not reported before.

## Case presentation

**Figure 1:**
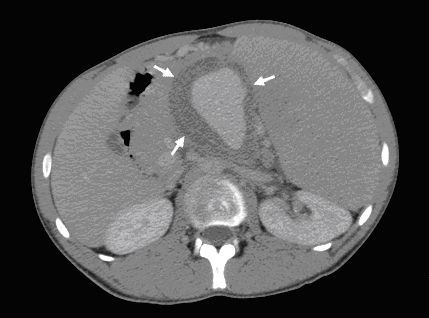
**CT scan of the abdomen showed massive splenomegaly and a solid mass with hypodense area in the tail of the pancreas (arrows)**.

**Figure 2:**
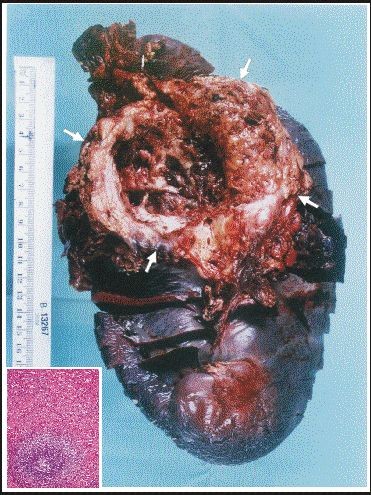
**Macroscopic pathology shows huge spleen measuring 23.5 × 15.5 × 6.3 cm and a cystic mass measuring 8.2 × 6.5 × 6.0 cm located in the tail of the pancreas adhered to the hilar region of the spleen (arrows)**. Microscopically, red pulp congestion and hyperplasia of the white pulp are shown in the left lower corner.

**Figure 3:**
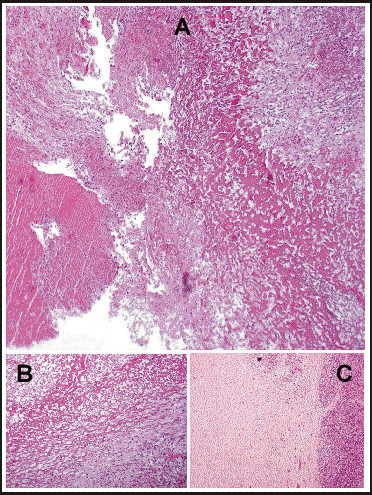
**Panoramic view of the IMT showing fibrin and cellular debris **(A). The inner part is formed by cellular area adjacent to necrotic and inflammatory material [Intermediate power magnification - Hematoxylin and eosin stain (B)]. The external area of the pancreatic tissue involved by myofibroblastic cells of the IMT [Low power magnification - Hematoxylin and eosin stain (C)].

Written informed consent was obtained from the patient for publication of this case report. A 19 years old male patient with no significant past medical history presented to emergency room with abdominal pain and fatigue without complains of anorexia, nausea, vomiting, weight loss, jaundice or fever. Physical examination revealed skin pallor, blood pressure 112/72, heart rate 92/min. Abdominal palpation revealed diffuse abdominal tenderness and splenomegaly 22 cm. The liver and regional lymph nodes were not palpable. The remaining physical examination was unremarkable. Computed tomography (CT) scan of the abdomen showed massive splenomegaly and a solid mass with hypodense area in the tail of the pancreas (Figure 1). No liver lesions or abdominal lymphadenopathy were identified. Blood analysis revealed hemoglobin 10.6 gr/dl, white blood cell were 7000/mm^3^, platelet count 271000/mm^3^. Other laboratory analysis including potassium, sodium, calcium, magnesium, phosphorus, blood urea nitrogen, creatinine, serum amylase, lipase, and liver chemistry were all within normal range. Five hours later, blood pressure dropped to 86/55 and reduction of hemoglobin level to 5.9 gr/dl was detected. These findings considered indications for urgent explorative laparotomy. Sudden massive bleeding may cause acute hypovolemic shock even without reduction in the hemoglobin level. The patient underwent an urgent explorative laparotomy. About 1.75 liters of blood were found in abdominal cavity. A large tumor arising from the tail of pancreas and local rupture of an enlarged spleen adjacent to the tumor were detected. Distal pancreatectomy and splenectomy were performed. The postoperative course was without any remarkable complications. Macroscopic pathology revealed a cystic mass measuring 8.2×6.5×6.0 cm in the tail of the pancreas and huge spleen measuring 23.5×15.5×6.3 cm (Figure 2). The pancreatic tumor was adhered to the hilar region of the spleen. The wall of the cystic mass was 1.4 cm. Microscopic pathology showed diffuse myofibroblastic proliferation of the wall of the cystic mass with a variable inflammatory component surrounded by pancreatic parenchyma (Figure 3). The patient has been followed for 6 years without any clinical or radiographic evidence of recurrence.

## Discussion

IMT is a histopathologic entity previously known as an inflammatory pseudotumor which was initially reported in 1990 in the pulmonary system [4]. Different names have been used to describe this entity, such as plasma cell granuloma, plasma cell pseudotumor, inflammatory fibroxanthoma, inflammatory pseudotumor and histiocytoma [5]. The histological features vary slightly from site to site, which may, at least in part, be related to differences in the phase of the lesion's development at the time of the detection. Representative features include the presence of a myofibroblastic proliferation and a varying degree of inflammatory infiltrates, mainly consisting of lymphocytes, histiocytes and plasma cells [6]. A number of the clinical and pathological features of IMT suggest the possibility that this lesion is more similar to a neoplasm than an inflammatory lesion [7]. Some investigators argue that IMT may be a true sarcoma and prefer the term inflammatory fibrosarcoma [7-9]. Whether IMT and inflammatory fibrosarcoma are actually the same tumor or different entities, it is remains controversial. Now, it is generally accepted that IMT is indeed a true neoplasm with a wide spectrum of histopathological behavior, varying from benign lesions to rare aggressive tumors [7]. Recently, inflammatory fibrosarcoma has become included in the spectrum of inflammatory myofibroblastic proliferations [10].

Although IMT occurs more frequently in the pulmonary system but it had been described in a wide variety of other organs [6]. In a clinicopathologic and immunohistochemical study of 84 cases of extrapulmonary IMT, the involved organs were intra-abdominal sites in 49 cases (58.4%), upper respiratory tract in 9 cases (10.7%), genitourinary tract in 8 cases (9.5%), trunk in 8 cases (9.5%), pelvis and retroperitoneum in 4 cases (4.8%), extremities in 3 cases (3.6%), and head and neck in 3 cases (3.6%) [11-13]. Furthermore, IMT has also been reported in the orbit [14], salivary glands [15], spleen [16-18], liver [19,20], urinary bladder and soft tissues [20,21], skin [22], kidneys [23], heart [24] and central nervous system [25].

IMT of the pancreas is rare. Only 27 cases of IMT located in the pancreas have been reported in English literature [5,6,26-43]. The age distribution of IMT of the pancreas resembled that of in pulmonary system ranging 2.5 to 70 years. IMT equally affects males and females. Commonly, the clinical presentation of IMT of the pancreas is a mass discovered incidentally by imaging investigations for other reasons. The presenting symptoms and signs of pancreatic IMT were abdominal pain (65.4%), unintentional weight loss (42.3%), jaundice (38.5%), palpable abdominal mass (26.9%) and anemia (23.0%). Associated clinical symptoms included fatigue (11.5%), fever (7.5%), anorexia (7.5%), nausea and vomiting (7.5%).

The presenting symptoms of the reported patient were abdominal pain and fatigue as a result of massively enlarged spleen and anemia due to substantial congestion of the spleen resulted from the location of the IMT in the tail of pancreas that cause a considerable obstruction of the blood drainage from the spleen. The abdominal pain and the anemia caused mainly by the huge palpable spleen rather than by the tumor itself. Anorexia, nausea, vomiting, weight loss or jaundice, were not included in the presenting symptoms of our patient. It was reported that one patient with IMT located in the body and the tail of the pancreas developed splenic vein thrombosis resulting in splenomegaly, thrombocytopenia and upper gastrointestinal hemorrhage from isolated gastric varices [31]. The present patient with IMT located in the tail of the pancreas developed massively enlarged spleen as a result of mechanical obstruction caused by the tumor itself and complicated with abdominal bleeding due to spontaneous splenic rupture. No thrombosis of splenic vessles was found and no gastric varices or upper gastrointestinal hemorrhage were detected. Surprisingly, also no thrombocytopenia or leukopenia, were observed.

The tumor was located in the head of the pancreas in 57.7% of cases, whereas it was found in the body and the tail of the pancreas in 42.3%. IMT of the pancreas has a tendency to be a larger in size than that in the pulmonary system at the time of diagnosis ranging 1.5-13 cm [5,11]. The size of the IMT in the presented patient was 8.2 × 6.5 × 6.0 cm. Almost all patients underwent exploratory laparotomy and surgical resection. However, correct diagnoses were not made in any of these patients including the presented patient before pancreatic resection, even with an intraoperative frozen section biopsy.

Complete surgical resection is the treatment of choice for IMT without any need of chemotherapy and radiation therapy [24,44,45]. The prognosis of IMT is generally good, with only a rare incidence of malignant transformation [44]. However, a significant recurrence rate of 25% was reported [11]. IMT of the retroperitoneum has susceptibility for more aggressive behavior with multiple recurrences [44]. It was suggested that the presence of atypia, ganglion-like cells and p53 expression may suggest more aggressive behavior [46,47]. These lesions may be indistinguishable from inflammatory fibrosarcoma due to a high degree of clinical and morphological overlap [44]. Radiation therapy [9,48,49], immunosuppressive therapy [50] and chemotherapy with or without combined radiation therapy [11,44] had been considered in the management of aggressive IMT or inflammatory fibrosarcoma.

Because of a higher recurrence rate, approaching 25% in extrapulmonary IMT, our patient has been closely followed with no clinical or radiographic evidence of recurrence for 6 years following surgery.

## Conclusion

Inflammatory myofibroblastic tumor of the tail of pancreas should be included in the differential diagnosis of the etiological causes of massively enlarged spleen and spontaneous splenic rupture.

## Competing interests

The authors declare that they have no competing interests.

## Authors' contributions

KH participated actively in the diagnosis process, following up the patient, preparing, writing and revising the literature and the manuscript. HC is the pathologist that carried out the pathological diagnosis, edited and revised the figures' legends. FH participated actively in preparing, writing, editing, printing and revising the manuscript. HS participated actively in following up the patient, reviewing the literature, preparing, editing and revising the manuscript. All authors read and approved the final manuscript.
